# Breast density as a determinant of interval cancer at mammographic screening

**DOI:** 10.1038/sj.bjc.6601548

**Published:** 2004-01-20

**Authors:** S Ciatto, C Visioli, E Paci, M Zappa

**Affiliations:** 1 Centro per lo Studio e la Prevenzione Oncologica, Viale Volta 171, 50131 Firenze, Italy

**Keywords:** breast cancer, diagnosis, mammography, screening, interval cancer

## Abstract

The association of breast density (% of breast volume involved by fibro-glandular densities) with the risk of interval cancer (IC) was investigated by reviewing a consecutive series of 346 cancers detected at screening (SDC) during 1996–1999 and of 90 ICs, reported as negative in the same period and diagnosed in the following 2 years, and comparing them to a random sample of 360 healthy controls. The probability of IC was significantly associated with breast density, whatever grouping (0/1–25/26–74/>74%; 0–25/26–60/61–74/>74%; 0–25/26–74/>74%) was considered (*χ*^2^=30.67–34.08, *P*<0.<0.01): 27.8% of all ICs were classified in the >74% density class, as compared to 7% of SDC and 5% of healthy controls. No significant association to IC was observed for Wolfe pattern (P2/Dy *vs* N1/P1: *χ*^2^=0.30, *P*=0.960), number of used mammographic views (single oblique *vs* oblique+craniocaudal: *χ*^2^=0.02, *P*=0.90) or screening round (first *vs* repeat: *χ*^2^=1.41, *P*=0.23). Multivariate analysis confirmed the independent association of breast density to IC, the highest risk being observed for >74% density class (OR *vs* 0% class=13.4, 95% CI 2.7–65.6, OR *vs* all other density classes=5.1, 95% CI 2.6–10.0). Age showed an independent association too, older women having a lower risk of IC (OR=0.52 95% CI 0.3–09). Breast density (>74%) resulted as being a major determinant of IC. Special screening protocols (shorter rescreening interval, routine use of ultrasonography) might be suggested for these subjects in order to improve screening sensitivity and efficacy.

Mammographic screening is effective in reducing breast cancer mortality (Wald *et al*, 1993), although its sensitivity is not high. Based on interval cancer (IC) proportional incidence, biennal screening sensitivity has been estimated to be as low as 75% (Paci *et al*, 1990; Zappa *et al*, 2002). Thus, efforts should be made to reduce IC frequency, and determining the causes of screening faults is a baseline condition for further action.

At retrospective review (Egan and Mosteller, 1977; Ciatto *et al*, 1995a,1995b), most ICs are classified as ‘occult’ or ‘true interval’, a relevant proportion as ‘minimal sign’ and a minority as ‘screening error’. The corresponding frequencies determined for the Florence District Programme were 61.9, 26.1 and 11.9%, respectively (Ciatto *et al*, 1995a,1995b). Breast radiological density may have a masking effect on small cancer lesions; it is likely to be a determinant of false-negative mammography (Peeters *et al*, 1989), and might be associated with IC, particularly in cases reviewed as ‘occult’. Other variables, such as age (Wald *et al*, 1993), use of single or double view (Bryan *et al*, 1995) and use of single or double reading (Ciatto *et al*, 1995a,1995b) have been shown to be associated with mammography sensitivity and might also be associated to IC.

In Florence District, mammographic screening has been ongoing at the Centro per lo Studio e la Prevenzione Oncologica (CSPO) since 1970, being extended to Florence City in 1990. Screening efficacy has been demonstrated by means of a case–control study (Palli *et al*, 1986), and the proportional IC rate in women aged 50–69 years has been reported to be 18% (95% CI 10–24) and 42% (95% CI 29–59) in the first or second year of the interval, respectively: the sensitivity of biennal screening, estimated on the basis of proportional IC incidence, was 75% (Paci *et al*, 1990).

In the present study, a consecutive series of IC was compared to screen-detected cancers (SDC), and the association of breast density and other variables to IC was determined by univariate and multivariate analysis. Parameters significantly and independently associated to IC were identified, and the related implications on screening modalities were discussed.

## MATERIAL AND METHODS

The clinical records of all women attending the Florence City screening programme from 1996 to 1999 were considered for the study, and three groups of subjects were selected:

- all invasive ICs, diagnosed within 2 years after a negative screening mammogram, were identified by linkage with CSPO clinical archives (CSPO is the main breast clinic in the District, diagnosing over 80% of all incident breast cancers in the area) and with the Tuscany Cancer Registry, covering the whole district area since 1985.
- all invasive SDC detected in the study period, as recorded in the screening programme database
- a random sample of healthy subjects, with a negative screening mammogram reported in the study period and at further biennal screening, matched by age and screening year to IC according to a 1 : 4 ratio

For all these subjects, the original screening mammograms were blindly reviewed by one of us (SC) and the following variables were recorded:

- number of mammographic views: according to the local screening protocol, radiologists decide whether single- (mediolateral oblique) or double-view (mediolateral oblique+craniocaudal) mammography has to be performed at further screening, single view being recommended for ‘low-density’ breasts. At further screening, computer-produced lists with indication of the number of views to be taken are available to the radiographer. First screening mammograms, or mammograms for which the indication on the number of views is not available, are always taken in two views:
- Wolfe's parenchymal pattern, determined according to the original classification (Wolfe, 1976) by one of us (SC) and
- breast density, calculated as the proportion of breast volume occupied by fibro-glandular density. Cases were displayed on a rotating viewer and classified directly by one of us (SC) according to percentage breast density (0, 1–25, 26–74 and >74% density classes).
- IC type: IC had been previously classified by two expert CSPO radiologists as (a) screening error, (b) minimal sign and (c) radiologically occult, in accordance to EC guidelines (Perry *et al*, 2001).

The distribution of studied variables among SDC, IC and healthy controls was first studied at univariate analysis: differences were checked with the *χ*^2^ test, and statistical significance was set at *P*<0.05. Multivariate analysis of the association of different variables to IC was performed by means of multiple logistic regression using the Stata 7.0 software.

## RESULTS

The study considers (a) all (*N*=346) SDC detected during 1996–1999, (b) all (*N*=90) subjects with a negative screening mammogram in the same period and developing an IC in the following 2 years and (c) a random sample of 360 subjects, assumed as healthy controls (CO), with a negative mammography during 1996–1999 and at further screening, after 2 years. Table 1

**Table 1:** Distribution of studied cases by age

Age (years)	***CO (%)***	***IC (%)***	***SDC (%)***
50–59	181 (50.3)	48 (53.3)	138 (39.9)
60–69	140 (38.9)	35 (38.9)	175 (50.6)
70+	39 (10.8)	7 (7.8)	33 (9.5)
			
Total	360 (100)	90 (100)	346 (100)
			
Median age	59.2	59.3	62.4

SDC=screen-detected cancer; IC=interval cancers; CO=healthy controls.

shows the distribution of studied cases by age: no difference was evident as to age grouping (*χ*^2^=0.7, *P*=0.674), average or median age when comparing cancers and negative controls, whereas a minor, not statistically significant, difference was evident between SDC and ICs (*χ*^2^=5.3, *P*=0.07).

Table 2

**Table 2:** Distribution of cases according to studied mammographic features

	**CO (%)**	**IC (%)**	**SDC (%)**
Wolfe pattern			
N1	41 (11.4)	6 (7.5)	27 (8.1)
P1	174 (48.3)	28 (35.0)	98 (29.4)
P2	120 (33.3)	48 (48.7)	176 (52.9)
Dy	25 (7.0)	9 (8.8)	32 (9.6)
			
Density (I)			
0%	36 (10.0)	2 (2.2)	26 (7.8)
1–25%	173 (48.1)	29 (32.2)	96 (28.8)
26–74%	133 (36.9)	34 (37.8)	187 (56.2)
>74%	18 (5.0)	25 (27.8)	24 (7.2)
			
Density (II)			
0–25%	209 (58.1)	31 (34.4)	122 (36.6)
26–60%	97 (26.9)	24 (26.7)	138 (41.4)
61–74%	36 (10.0)	10 (11.1)	49 (14.7)
>74%	18 (5.0)	25 (27.8)	24 (7.2)
			
Density quartiles			
25%	10	25	20
50%	25	50	40
75%	50	75	60
			
Mammographic views			
1	240 (66.7)	51 (56.7)	190 (57.4)
2	120 (33.3)	39 (43.3)	141 (42.6)
			
Screening round			
First	14 (3.9)	11 (12.2)	63 (18.2)
Repeat	346 (96.1)	79 (78.8)	283 (81.8)

SDC=screen-detected cancer; IC=interval cancers; CO=healthy controls.

shows the association of studied variables to cancer and/or IC. A significant difference in Wolfe pattern distribution (excess of P2/DY patterns in cancers) was observed when comparing cancers and negative controls (*χ*^2^=14.3, *P*=0.002), whereas no significant difference was evident between SDC and ICs (*χ*^2^=0.3, *P*=0.96). Whatever grouping of breast density classes was considered (0/1–25/26–74/>74%; 0–25/26–60/61–74/>74%; 0–25/26–74/>74%), a significantly different distribution (excess density in cancers or in IC) was evident when comparing cancers to negative controls (*χ*^2^=46.6–48.2, *P*<0.01) or ICs to SDC (*χ*^2^=30.6–34.0, *P*<0.01). Overall, 27.8% of ICs were classified in the >74% density class, as compared to 7% of SDC and 5% of healthy controls. The distribution of cases by density quartiles confirms the density pattern of the three groups: 75% of healthy controls had a breast density <50% as compared to 60 and 75% for SDC and ICs, respectively. A single oblique view was more frequent, although with borderline significance, among healthy controls as compared to cancers (*χ*^2^=3.15, *P*=0.08), whereas no significant difference was evident when comparing SDC and ICs (*χ*^2^=0.02, *P*=0.90). As far as the attended round (first or repeat) was concerned, the proportion of ICs and SDC at first screening was higher as compared to healthy controls (*χ*^2^=33.0, *P*=10^−6^), whereas no statistically significant difference was evident between SDC and ICs (*χ*^2^=1.4, *P*=0.23).

The distribution of SDC and ICs according to the two studied classifications (Wolfe's pattern or percentage density) is shown in Table 3

**Table 3:** Distribution of cancer cases (screen detected and interval) according to Wolfe's and proportional density classification

	**Proportional density**				
**Wolfe pattern**	**0**	**1–25%**	**26–74%**	**>74%**	**Total**
N1	28	5	0	0	33 (7.8%)
P1	0	116	10	0	126 (29.8%)
P2	0	4	187	33	224 (53.0%)
DY	0	0	24	16	40 (9.4%)
					
Total	28 (6.6%)	125 (29.6%)	222 (52.5%)	49 (11.6%)	423 (100%)

. On a two-grade scale (N1-P1 *vs* P2-Dy patterns, and 0–25 *vs* 26–100% density), the association between Wolfe's high-grade (P2-Dy) and dense (>25%) breasts was quite evident, with 96.6% of cases falling in the N1-P1/0–25% or P2-Dy/26–100% subgroups.

Table 4

**Table 4:** Results of multivariate analysis of the association of different variables to detection as interval cancer

**Variable**	**Odds ratio**	**Confidence limits**	***P*-value**
*Age (years)*			
50–59	1^a^		
60–69	0.52	0.30–0.90	0.02
			
*Density*			
0%	1^a^		
1–25%	4.29	0.95–19.4	0.06
25–74%	2.28	0.51–10.2	0.28
>74%	13.38	2.73–65.6	<.001
			
*Mammographic views*			
1	1^a^		
2	0.76	0.41–1.44	0.41
			
*Screening round*			
First	1^a^		
Repeat	1.85	0.81–4.2	0.14

a Reference category.

shows the results of multivariate analysis of the association of different variables to IC. Wolfe's parenchymal pattern did not enter the model, as it showed a high collinearity with breast density, and the latter, fitting best at univariate analysis, was used for logistic regression. Modification effects were tested but not statistically significant. Older women had a lower probability of developing IC (OR=0.52 95% CI 0.3–09); breast density showed a significant association to IC, particularly for the >75% density class (OR=13.4, 95% CI 2.7–65.6). Attendance at repeat rather than at first screening round was also associated to IC, although not at a significant level (OR=1.85, 95% CI 0.8–4.2), whereas no significant association was evident for the number of mammographic views used.

Table 5

**Table 5:** Classification of interval cancers according to proportional breast density and error type

	**Proportional density**				
**Error type**	**0**	**1–25%**	**25%–74%**	**>74%**	**Total**
Screening error	1	11	6	1	19 (21.4%)
Minimal signs	1	8	14	8	31 (34.8%)
Occult	0^a^	10	14	15	39 (43.8%)

a One case not available for film review.

shows the distribution of IC by density class and by the type of screening error. The two variables were significantly associated (*χ*^2^=18.09, *P*=0.03) as most IC classified as occult occurred in dense breasts, whereas most screening errors occurred in low-density breasts. No association to density was observed among cases classified as minimal signs.

## DISCUSSION

This study was aimed at identifying specific mammographic features associated to IC, thus allowing the selection of subgroups for alternative, more aggressive screening regimens (e. g. increased screening frequency, routine ultrasonography).

Age was significantly associated to IC, a finding already reported in screening studies (Paci *et al*, 1990; Wald *et al*, 1993). Differences in breast density are the most likely explanation for the age-related sensitivity of mammography, but the association observed in the present study persisted after adjustment for breast density. Nevertheless, the magnitude of such an association was too small to justify a special screening regimen in younger women within subjects eligible for screening (age 50–69 years).

Wolfe's parenchymal pattern was associated to breast cancer, confirming several previous reports on this subject (Wolfe, 1976). Wolfe's pattern classification has been questioned for its inter- and intraobserver inconsistency. An analysis of intraobserver variability performed within this study on a subset of 300 cases on a two-grade scale (N1/P1 *vs* P2/Dy patterns) gave an absolute concordance rate of 85%. Wolfe's Pattern distribution for SDC and healthy controls in this study (P2-Dy; SDC=62.5, CO (age 50–64 years)=44.5%) was comparable to that observed (P2-Dy: SD=67%, CO=59%) in a case–control study performed at the Cambridge Screening Programme (Sala *et al*, 1998), with minor differences possibly accounted for by differences in hormone replacement therapy (HRT) use, or other factors associated with dense parenchyma (e.g. diet). However, Wolfe pattern was not significantly associated to the risk of IC, and thus was of no value in selecting high-risk subjects to special screening regimens.

Proportional breast density attribution is also subjective, and exposed to inter- and intraobserver variability. An analysis of intraobserver variability performed within this study on a subset of 300 cases on a two-grade scale (0–25 *vs* >25% density) gave an absolute concordance rate of 92%. To avoid such variability of eye judgement, breast density might be quantitatively determined by a computer on digitised films, but that would imply technology, which is not currently available everywhere. Proportional breast density is a purely quantitative parameter, directly associated to the probability of a ‘masking effect’ (Egan and Mosteller, 1977), whereas Wolfe's classification is also based on morphological criteria predictive of dysplasia (Ciatto and Zappa, 1993). The two classifications are different, and this justifies our findings at univariate analysis, which revealed a significant association to IC risk only for proportional density and not for Wolfe's pattern.

Hormone replacement therapy use has been reported to increase breast density (Berkowitz and Gatewood, 1990) and potentially affect screening efficacy (Ma *et al*, 1992). Hormone replacement therapy was not recorded in the present study, but a previous study in the adjacent district of Siena reported a 27.7% HRT use in postmenopausal screening attenders (age 50–59 years), and confirmed an increased probability of dense breast in HRT users (density >25%=+17%, density >50%=+7%) as compared to nonusers (Ciatto *et al*, 2001).

The number of mammographic views used has been reported to affect mammography sensitivity, suggesting a higher frequency of IC after a single-view as compared to two-view mammography screening (Bryan *et al*, 1995). This was not evident in the present study. The fact that the single view in our study was limited to repeat screening and to selected subjects with nondense breast might at least partially explain our findings.

In conclusion, the present study suggests a strong association of breast density to screening sensitivity that is reduced in dense breasts, a finding also reported in other studies (Ciatto and Zappa, 1993; Sala *et al*, 1998). High breast density (>74%) was reported in 28% of ICs as compared to 7.2% of SDC or 5% of healthy controls. At multivariate analysis, the risk of IC (adjusted for others covariates) associated to >74% density was significantly higher (odd ratio=5.1 : 1, 95% CI 2.6–10.0) as compared to subjects with lower density values. Straightforward calculations based on IC breast density would suggest that up to 22% of IC might be prevented if adopting a special screening regimen in subjects with >74% density would achieve the same sensitivity as in the general population. These findings suggest the opportunity to investigate alternative screening regimens (e.g. reduced rescreening interval, routine use of ultrasound) to maximise sensitivity in these subjects.
